# “THERE’S A LOT OF FACTORS”: UTILIZING I-POETRY TO UNDERSTAND VACCINE-HESITANT DECISION MAKING

**DOI:** 10.1093/geroni/igad104.2842

**Published:** 2023-12-21

**Authors:** Bryce Van Vleet, Heather Fuller, Andrea Huseth-Zosel, Paul Carson

**Affiliations:** North Dakota State University, Fargo, North Dakota, United States; North Dakota State University, Fargo, North Dakota, United States; North Dakota State University, Fargo, North Dakota, United States; North Dakota State University, Fargo, North Dakota, United States

## Abstract

Despite higher vaccinations rates as compared to other age groups, many US older adults remain under-vaccinated against common, preventable diseases leaving them at increased risk of serious illness, hospitalization, or death posed from diseases including shingles, pneumonia, influenza, and novel coronavirus. This study investigates the underlying reasons older adults have for not getting vaccinated. Eight older adults (Mean Age = 73.86) selected from a larger interview study conducted in North Dakota were identified as being vaccine hesitant as indicated by receiving two or fewer vaccines from a list of five common vaccines including influenza, shingles, pneumococcal, and initial and booster doses of the COVID-19 vaccine. Participants were asked, “How do you decide about whether or not to get a specific vaccine?” Their open-ended responses were analyzed using the I-poetry qualitative methodology which combined participants’ “I” statements into a poem for all eight participants that reflected their underlying motivations of vaccine hesitancy. This poem was structured in stanzas to highlight and convey overarching themes including: practical barriers, vaccine-alternatives, and skepticism. Practical barriers included allergies and concerns about side effects. Vaccine-alternatives highlighted a belief in natural immunity, vitamins, and exercise. Skepticism was directed at government agencies and the efficacy of a vaccine. These findings highlight potential benefits to providing medical professionals with first-person narratives from vaccine hesitant older adults which could help clarify sources of unease. Implications can be drawn for better understanding this at-risk population and developing tailored and effective interventions to bolster older adults’ vaccine uptake.

